# The genome sequence of the Rustic Shoulder-knot, *Apamea sordens* (Hufnagel, 1766)

**DOI:** 10.12688/wellcomeopenres.18712.1

**Published:** 2023-02-02

**Authors:** Douglas Boyes, University of Oxford and Wytham Woods Genome Acquisition Lab, Darwin Tree of Life Barcoding collective, Wellcome Sanger Institute Tree of Life programme, Wellcome Sanger Institute Scientific Operations: DNA Pipelines collective, Tree of Life Core Informatics collective, Peter W.H. Holland, Darwin Tree of Life Consortium

**Affiliations:** 1 UK Centre for Ecology and Hydrology, Wallingford, Oxfordshire, UK; 2 Department of Biology, University of Oxford, Oxford, Oxfordshire, UK

**Keywords:** Apamea sordens, Rustic Shoulder-knot, genome sequence, chromosomal, Lepidoptera

## Abstract

We present a genome assembly from an individual male *Apamea sordens* (the Rustic Shoulder-knot; Arthropoda; Insecta; Lepidoptera; Noctuidae). The genome sequence is 614 megabases in span. The whole assembly is scaffolded into 31 chromosomal pseudomolecules, including the assembled Z sex chromosome. The mitochondrial genome has also been assembled and is 16.3 kilobases in length.

## Species taxonomy

Eukaryota; Metazoa; Ecdysozoa; Arthropoda; Hexapoda; Insecta; Pterygota; Neoptera; Endopterygota; Lepidoptera; Glossata; Ditrysia; Noctuoidea; Noctuidae; Noctuinae; Apameini; *Apamea*; *Apamea sordens* (Hufnagel, 1766) (NCBI:txid689061).

## Background

*Apamea sordens* (the Rustic Shoulder-knot) is a moth in the family Noctuidae found commonly in grasslands, gardens, farmland and woodland rides in the UK. The moth is found across the Palaearctic as far east as China and Japan, and also occurs in North America ( *National Biodiversity Atlas*). The pattern and position of cross lines, marginal marks and circles (orbicular and reniform stigma) on the adult forewings are very similar to those of several closely related species, which can make distinguishing members of the genus *Apamea* difficult. The consistency of the markings suggests these moths share conserved patterning mechanisms during wing development, in an analogous way to the well-studied nymphalid ground plan of many butterflies ( Nijhout, 1994; Schwanwitsch, 1929). *A. sordens* is distinguished from its close relatives by its pale brown, silvery appearance, and a diagnostic forked black streak at the base of each forewing (the ‘shoulder-knot’). The specific name *sordens*, meaning ‘dirty’, does not do justice to this delicately patterned moth ( Maitland Emmet, 1991).

In the south of the UK, the adult moth is usually on the wing one to two weeks earlier than other *Apamea* species, commencing in mid-May and seen until mid-June (PWHH, pers. obs.). Larvae of *A. sordens* feed on various grasses, overwintering as a larva in the UK and pupating in early spring ( Robinson *et al.*, 2010; Waring & Townsend, 2017). The species has been reported as an agricultural pest in several countries including Kazakhstan, Iran and Georgia, with larvae continuing to feed on cereal crops after harvest ( Hashemi *et al.*, 1995; Keburia *et al.*, 2010; Shek & Evdokimov, 1972). Availability of a genome sequence would facilitate future research into pest control strategies and into fundamental biological questions such as the molecular basis of wing patterning.

The genome of *A. sordens* was sequenced as part of the Darwin Tree of Life Project, a collaborative effort to sequence all named eukaryotic species in the Atlantic Archipelago of Britain and Ireland. Here we present a chromosomally complete genome sequence for *A. sordens*, based on the ilApaSord1 specimen from Wytham Woods, Berkshire, UK.

## Genome sequence report

**Figure 1.**
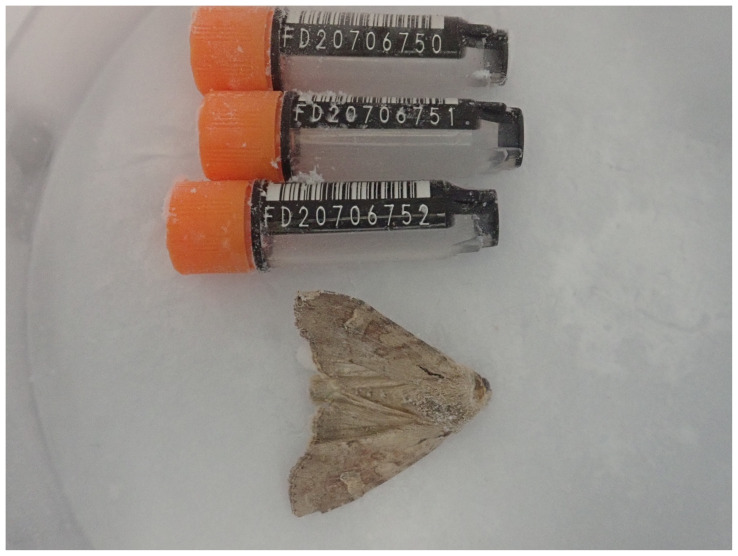
Photograph of the *Apamea sordens* (ilApaSord1) specimen used for genome sequencing.

The genome was sequenced from one male *A. sordens* specimen ( Figure 1) collected from Wytham Woods, Berkshire, UK (latitude 51.77, longitude –1.32). A total of 38-fold coverage in Pacific Biosciences single-molecule HiFi long reads was generated. Primary assembly contigs were scaffolded with chromosome conformation Hi-C data. Manual assembly curation corrected five missing or mis-joins and removed two haplotypic duplications, reducing the scaffold number by 8.82%.

**Figure 2.**
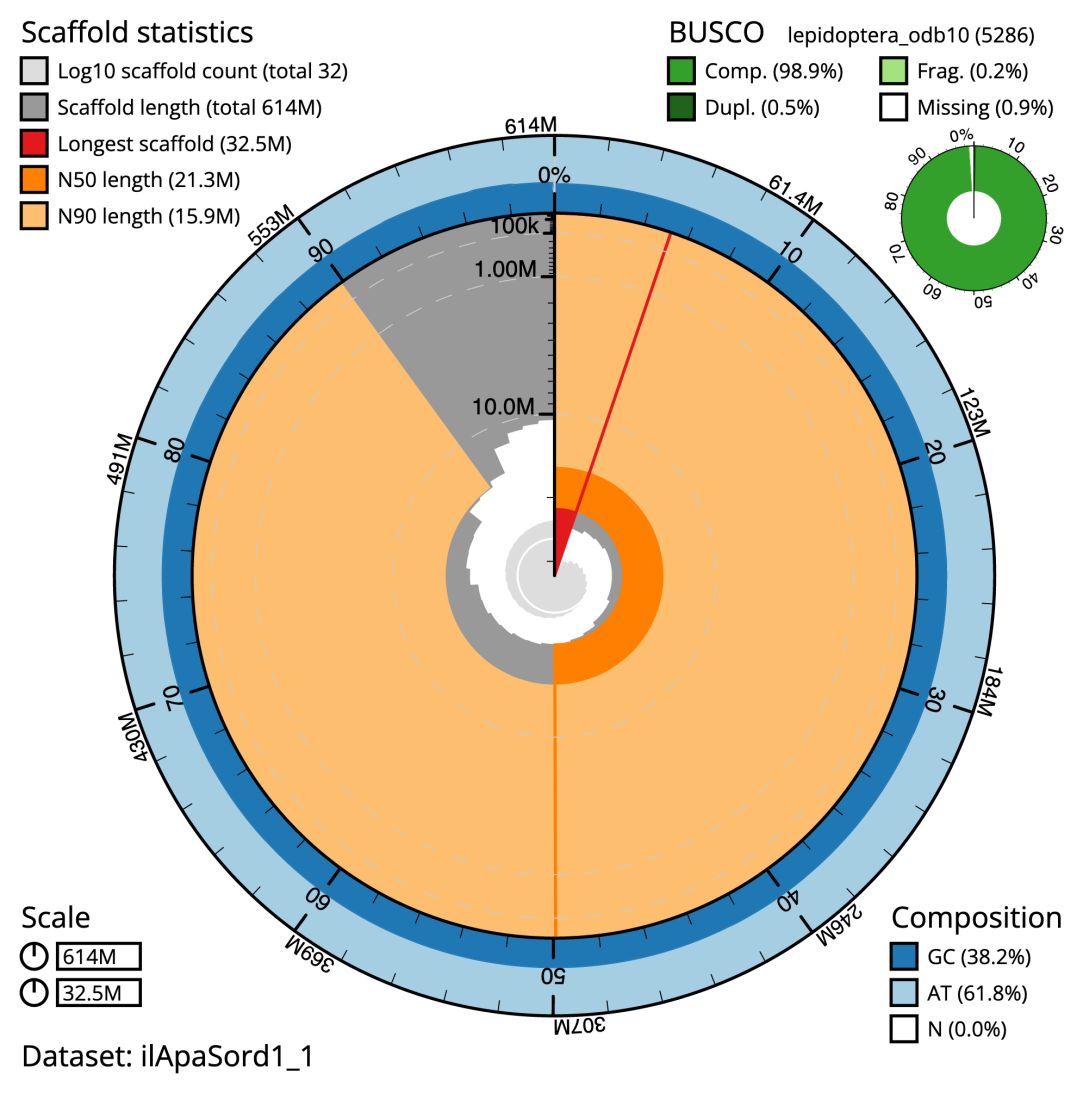
Genome assembly of *Apamea sordens*, ilApaSord1.1: metrics. The BlobToolKit Snailplot shows N50 metrics and BUSCO gene completeness. The main plot is divided into 1,000 size-ordered bins around the circumference with each bin representing 0.1% of the 614,329,545 bp assembly. The distribution of scaffold lengths is shown in dark grey with the plot radius scaled to the longest chromosome present in the assembly (32,512,628 bp, shown in red). Orange and pale-orange arcs show the N50 and N90 chromosome lengths (21,259,203 and 15,914,481 bp), respectively. The pale grey spiral shows the cumulative scaffold count on a log scale with white scale lines showing successive orders of magnitude. The blue and pale-blue area around the outside of the plot shows the distribution of GC, AT and N percentages in the same bins as the inner plot. A summary of complete, fragmented, duplicated and missing BUSCO genes in the lepidoptera_odb10 set is shown in the top right. An interactive version of this figure is available at https://blobtoolkit.genomehubs.org/view/ilApaSord1.1/dataset/ilApaSord1_1/snail.

The final assembly has a total length of 614.3 Mb in 31 sequence scaffolds with a scaffold N50 of 21.3 Mb ( Table 1). The whole assembly sequence was assigned to 31 chromosomal-level scaffolds, representing 30 autosomes and the Z sex chromosome. Chromosome-scale scaffolds confirmed by the Hi-C data are named in order of size ( Figure 2– Figure 5; Table 2). The mitochondrial genome was also assembled. The assembly has a BUSCO v5.3.2 ( Manni *et al.*, 2021) completeness of 98.4% using the lepidoptera_odb10 reference set. Evaluation of the assembly shows a consensus quality value (QV) of 72.1 and *k*-mer completeness of 100%. While not fully phased, the assembly deposited is of one haplotype. Contigs corresponding to the second haplotype have also been deposited.

**Table 1.** Genome data for *Apamea sordens*, ilApaSord1.1.

Project accession data		
Assembly identifier	ilApaSord1.1	
Species	*Apamea sordens*	
Specimen	ilApaSord1	
NCBI taxonomy ID	689061	
BioProject	PRJEB54051	
BioSample ID	SAMEA10166870	
Isolate information	male; thorax (PacBio sequencing), head (Hi-C)	
Assembly metrics *		*Benchmark*
Consensus quality (QV)	72.1	*≥ 50*
*k*-mer completeness	100%	*≥ 95%*
BUSCO **	C:98.9%[S:98.4%,D:0.5%], F:0.2%,M:0.9%,n:5,286	*C ≥ 95%*
Percentage of assembly mapped to chromosomes	100%	*≥ 95%*
Sex chromosomes	Z chromosome	*localised* *homologous pairs*
Organelles	Mitochondrial genome assembled	*complete single* *alleles*
Raw data accessions		
PacificBiosciences SEQUEL II	ERR9924612	
Hi-C Illumina	ERR9930687	
Genome assembly		
Assembly accession	GCA_945859715.1	
*Accession of alternate haplotype*	GCA_945859755.1	
Span (Mb)	614.3	
Number of contigs	35	
Contig N50 length (Mb)	21.0	
Number of scaffolds	31	
Scaffold N50 length (Mb)	21.3	
Longest scaffold (Mb)	32.5	

* Assembly metric benchmarks are adapted from column VGP-2020 of “Table 1: Proposed standards and metrics for defining genome assembly quality” from ( Rhie *et al.*, 2021). ** BUSCO scores based on the lepidoptera_odb10 BUSCO set using v5.3.2. C = complete [S = single copy, D = duplicated], F = fragmented, M = missing, n = number of orthologues in comparison. A full set of BUSCO scores is available at https://blobtoolkit.genomehubs.org/view/ilApaSord1.1/dataset/ilApaSord1_1/busco.

**Figure 3.**
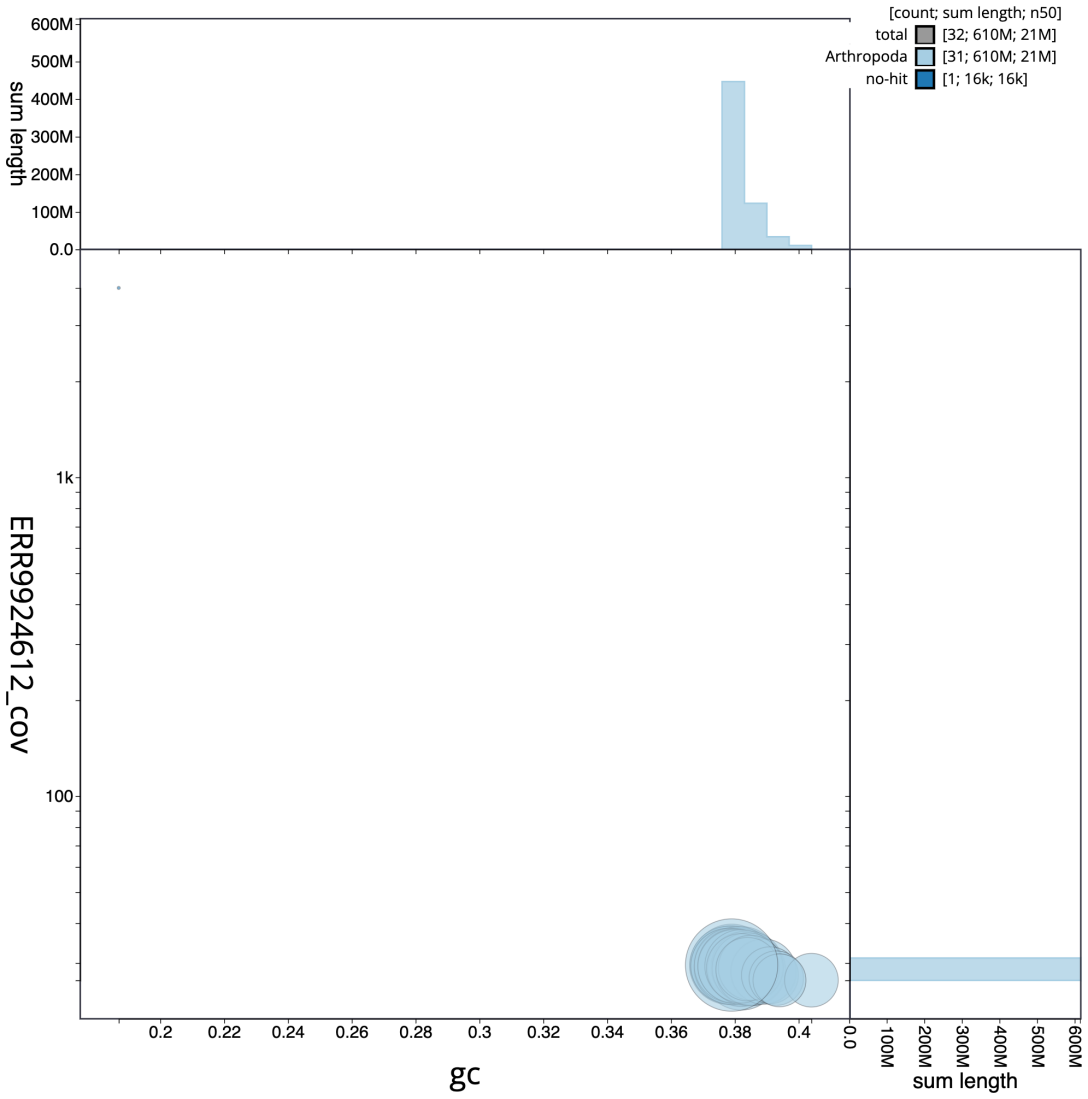
Genome assembly of *Apamea sordens*, ilApaSord1.1: GC coverage. BlobToolKit GC-coverage plot. Chromosomes are coloured by phylum. Circles are sized in proportion to scaffold length. Histograms show the distribution of scaffold length sum along each axis. An interactive version of this figure is available at https://blobtoolkit.genomehubs.org/view/ilApaSord1.1/dataset/ilApaSord1_1/blob.

**Figure 4.**
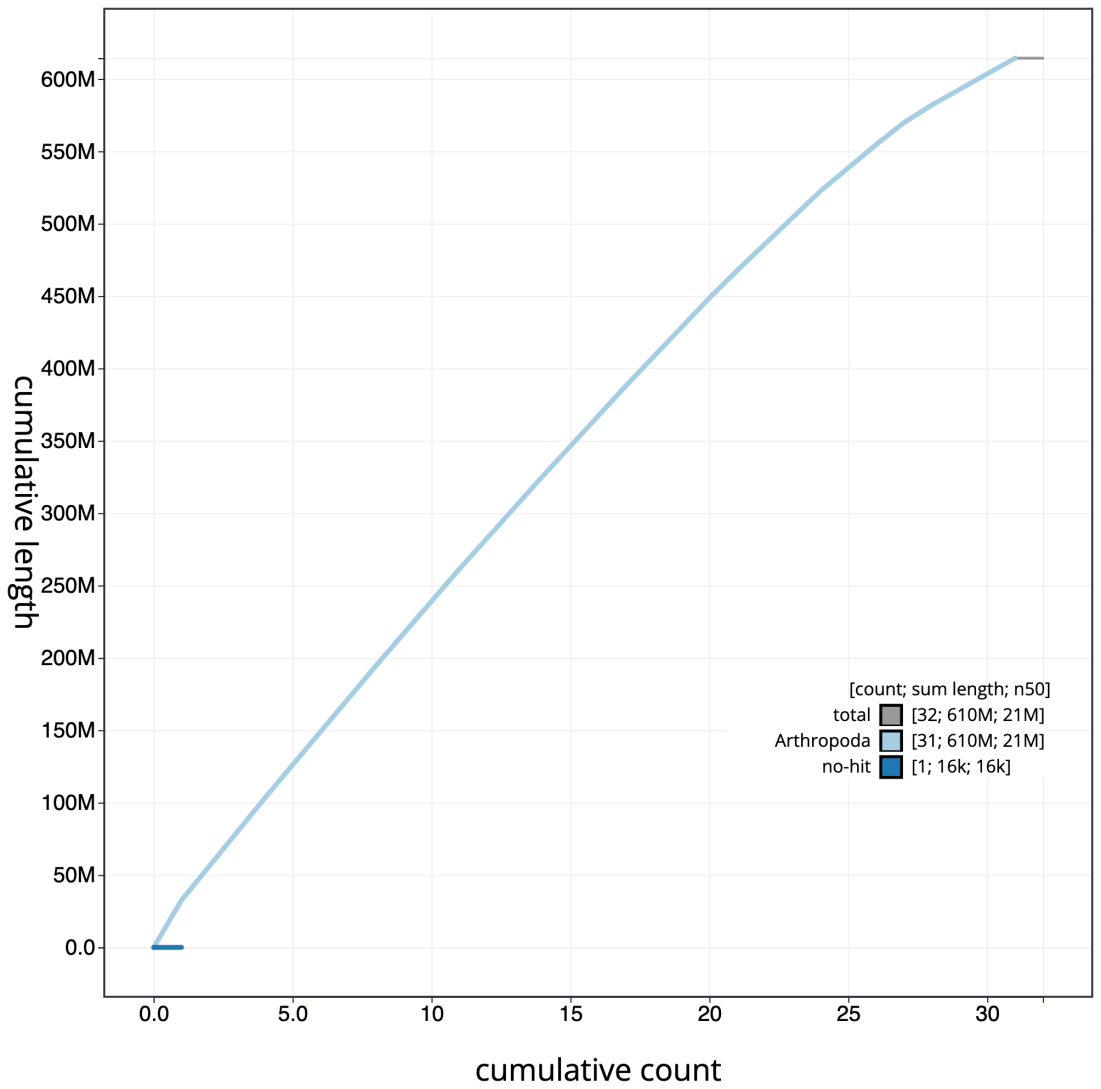
Genome assembly of *Apamea sordens*, ilApaSord1.1: cumulative sequence. BlobToolKit cumulative sequence plot. The grey line shows cumulative length for all scaffolds. Coloured lines show cumulative lengths of scaffolds assigned to each phylum using the buscogenes taxrule. An interactive version of this figure is available at https://blobtoolkit.genomehubs.org/view/ilApaSord1.1/dataset/ilApaSord1_1/cumulative.

**Figure 5.**
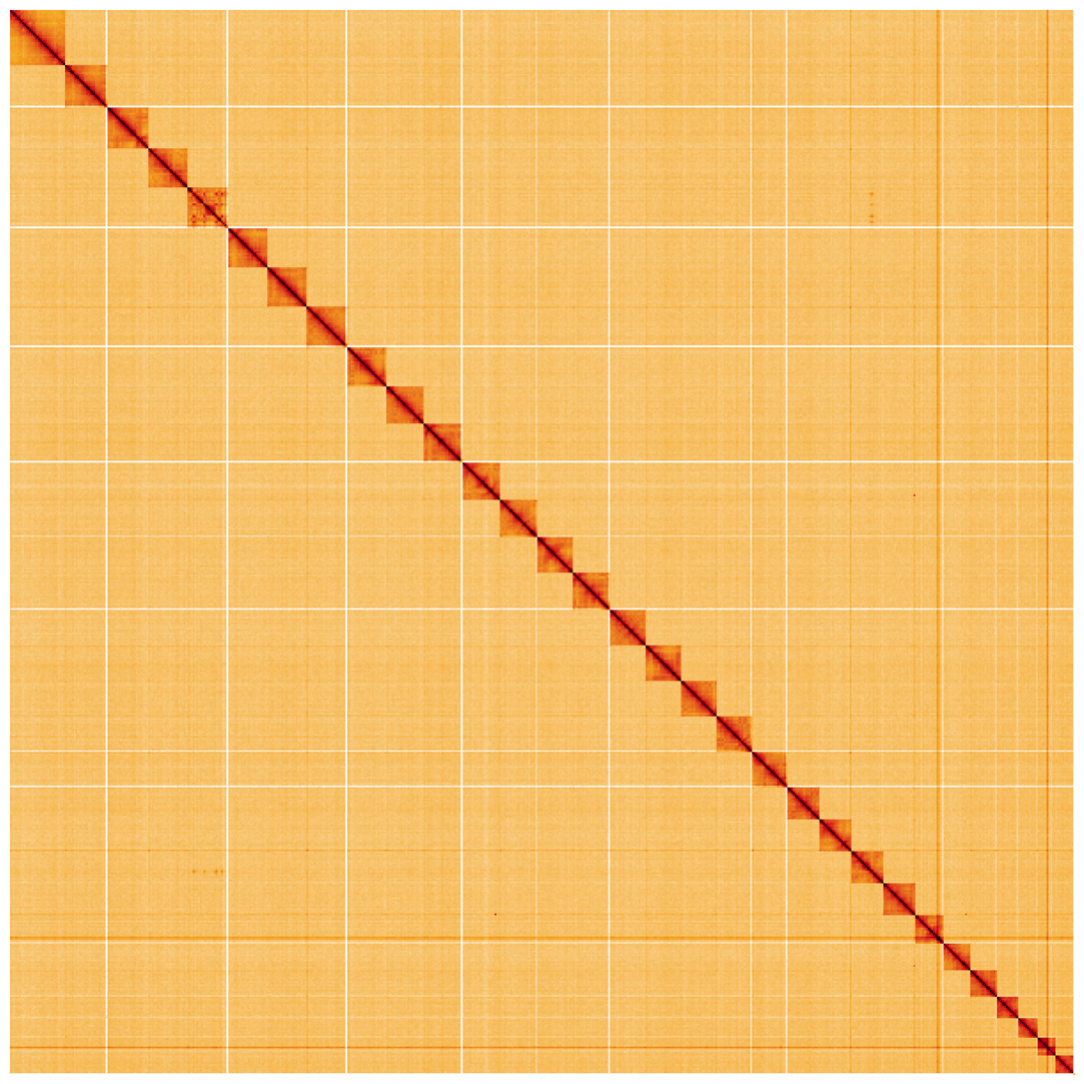
Genome assembly of *Apamea sordens*, ilApaSord1.1: Hi-C contact map. Hi-C contact map of the ilApaSord1.1 assembly, visualised using HiGlass. Chromosomes are shown in order of size from left to right and top to bottom. An interactive version of this figure may be viewed at https://genome-note-higlass.tol.sanger.ac.uk/l/?d=UY8hNZtsRzKBeffW2HEOow.

**Table 2.** Chromosomal pseudomolecules in the genome assembly of *Apamea sordens*, ilApaSord1.

INSDC accession	Chromosome	Size (Mb)	GC%
OX243986.1	1	24	37.9
OX243987.1	2	23.45	38.2
OX243988.1	3	23.22	37.9
OX243989.1	4	22.89	38.2
OX243990.1	5	22.88	37.8
OX243991.1	6	22.87	38.2
OX243992.1	7	22.86	38.2
OX243993.1	8	22.4	37.8
OX243994.1	9	22.39	37.9
OX243995.1	10	21.84	38.1
OX243996.1	11	21.57	37.8
OX243997.1	12	21.32	37.8
OX243998.1	13	21.26	37.9
OX243999.1	14	21.03	37.9
OX244000.1	15	20.94	38
OX244001.1	16	20.62	38
OX244002.1	17	20.48	38.3
OX244003.1	18	20.11	38.4
OX244004.1	19	20.04	38.2
OX244005.1	20	18.97	38.4
OX244006.1	21	18.41	38.5
OX244007.1	22	18.33	38.5
OX244008.1	23	18.19	38.2
OX244009.1	24	16.12	38.9
OX244010.1	25	15.91	38.5
OX244011.1	26	15.18	38.4
OX244012.1	27	12.14	39.1
OX244013.1	28	11.1	39.3
OX244014.1	29	10.79	40.4
OX244015.1	30	10.48	39.4
OX243985.1	Z	32.51	37.9
OX244016.1	MT	0.02	18.6

## Methods

### Sample acquisition and nucleic acid extraction

A male *A. sordens* specimen (ilApaSord1) was collected using a light trap from Wytham Woods, Berkshire, UK (latitude 51.77, longitude –1.32) by Douglas Boyes (University of Oxford). The sample was identified by Douglas Boyes and snap-frozen on dry ice.

DNA was extracted at the Tree of Life laboratory, Wellcome Sanger Institute. The ilApaSord1 sample was weighed and dissected on dry ice with tissue set aside for Hi-C sequencing. Thorax tissue was cryogenically disrupted to a fine powder using a Covaris cryoPREP Automated Dry Pulveriser, receiving multiple impacts. High molecular weight (HMW) DNA was extracted using the Qiagen MagAttract HMW DNA extraction kit. HMW DNA was sheared into an average fragment size of 12–20 kb in a Megaruptor 3 system with speed setting 30. Sheared DNA was purified by solid-phase reversible immobilisation using AMPure PB beads with a 1.8X ratio of beads to sample to remove the shorter fragments and concentrate the DNA sample. The concentration of the sheared and purified DNA was assessed using a Nanodrop spectrophotometer and Qubit Fluorometer and Qubit dsDNA High Sensitivity Assay kit. Fragment size distribution was evaluated by running the sample on the FemtoPulse system.

### Sequencing

Pacific Biosciences HiFi circular consensus DNA sequencing libraries were constructed according to the manufacturers’ instructions. DNA sequencing was performed by the Scientific Operations core at the WSI on the Pacific Biosciences SEQUEL II (HiFi) instrument. Hi-C data were also generated from head tissue of ilApaSord1 using the Arima v2 kit and sequenced on the Illumina NovaSeq 6000 instrument.

### Genome assembly

Assembly was carried out with Hifiasm ( Cheng *et al.*, 2021) and haplotypic duplication was identified and removed with purge_dups ( Guan *et al.*, 2020). The assembly was scaffolded with Hi-C data ( Rao *et al.*, 2014) using YaHS ( Zhou *et al.*, 2022). The assembly was checked for contamination as described previously ( Howe *et al.*, 2021). Manual curation was performed using HiGlass ( Kerpedjiev *et al.*, 2018) and Pretext ( Harry, 2022). The mitochondrial genome was assembled using MitoHiFi ( Uliano-Silva *et al.*, 2021), which performed annotation using MitoFinder ( Allio *et al.*, 2020). The genome was analysed and BUSCO scores generated within the BlobToolKit environment ( Challis *et al.*, 2020). Table 3 contains a list of all software tool versions used, where appropriate.

**Table 3.** Software tools and versions used.

Software tool	Version	Source
BlobToolKit	3.4.0	Challis *et al.*, 2020
Hifiasm	0.16.1-r375	Cheng *et al.*, 2021
HiGlass	1.11.6	Kerpedjiev *et al.*, 2018
MitoHiFi	2	Uliano-Silva *et al.*, 2021
PretextView	0.2	Harry, 2022
purge_dups	1.2.3	Guan *et al.*, 2020
YaHS	yahs-1.1.91eebc2	Zhou *et al.*, 2022

### Ethics/compliance issues

The materials that have contributed to this genome note have been supplied by a Darwin Tree of Life Partner. The submission of materials by a Darwin Tree of Life Partner is subject to the Darwin Tree of Life Project Sampling Code of Practice. By agreeing with and signing up to the Sampling Code of Practice, the Darwin Tree of Life Partner agrees they will meet the legal and ethical requirements and standards set out within this document in respect of all samples acquired for, and supplied to, the Darwin Tree of Life Project. Each transfer of samples is further undertaken according to a Research Collaboration Agreement or Material Transfer Agreement entered into by the Darwin Tree of Life Partner, Genome Research Limited (operating as the Wellcome Sanger Institute), and in some circumstances other Darwin Tree of Life collaborators.

## Data Availability

European Nucleotide Archive: *Apamea sordens* (rustic shoulder-knot). Accession number PRJEB54051; https://identifiers.org/ena.embl/PRJEB54051 ( Wellcome Sanger Institute, 2023). The genome sequence is released openly for reuse. The *Apamea sordens* genome sequencing initiative is part of the Darwin Tree of Life (DToL) project. All raw sequence data and the assembly have been deposited in INSDC databases. The genome will be annotated using available RNA-Seq data and presented through the Ensembl pipeline at the European Bioinformatics Institute. Raw data and assembly accession identifiers are reported in Table 1.

## Author information

Members of the University of Oxford and Wytham Woods Genome Acquisition Lab are listed here: https://doi.org/10.5281/zenodo.4789928.

Members of the Darwin Tree of Life Barcoding collective are listed here: https://doi.org/10.5281/zenodo.4893703.

Members of the Wellcome Sanger Institute Tree of Life programme are listed here: https://doi.org/10.5281/zenodo.4783585.

Members of Wellcome Sanger Institute Scientific Operations: DNA Pipelines collective are listed here: https://doi.org/10.5281/zenodo.4790455.

Members of the Tree of Life Core Informatics collective are listed here: https://doi.org/10.5281/zenodo.5013541.

Members of the Darwin Tree of Life Consortium are listed here: https://doi.org/10.5281/zenodo.4783558.
