# Supplementary material for: The genome sequence of the Rustic Shoulder-knot, Apamea sordens (Hufnagel, 1766)
Source: Wellcome Open Res. 2023 Feb 2;8:51. [Version 1] doi: 10.12688/wellcomeopenres.18712.1 (PMC10509607; doi:10.12688/wellcomeopenres.18712.1)
Supplement: Peer review history

# Reviewer report: Reviewer response for version 1

**DOI:** 10.21956/wellcomeopenres.20750.r66096

**Published:** 2023-09-19

**Authors:** Lin Liu

**Affiliations:** 1 Guangdong Provincial Key Laboratory of Insect Developmental Biology and Applied Technology, Guangzhou Key Laboratory of Insect Development Regulation and Applied Research, Institute of Insect Science and Technology, School of Life Sciences, South China Normal University, Guangzhou, China

The genome sequence of *Apamea sordens *was describe which is 614 megabases in span. The whole assembly is scaffolded into 31 chromosomal pseudomolecules, including the Z sex chromosome. The mitochondrial genome has also been assembled.

 The research method and data analysis are reasonable. Information is basically clear.

 Only one point on page 6 figure 3 "Chromosomes are coloured by phylum." Here, in figure 3, I can't get information of chromosomes. May the author provide more description in the legend.

Are sufficient details of methods and materials provided to allow replication by others?

Yes

Is the rationale for creating the dataset(s) clearly described?

Yes

Are the datasets clearly presented in a useable and accessible format?

Yes

Are the protocols appropriate and is the work technically sound?

Yes

Reviewer Expertise:

Genomics; function of genes

I confirm that I have read this submission and believe that I have an appropriate level of expertise to confirm that it is of an acceptable scientific standard.

# Reviewer report: Reviewer response for version 1

**DOI:** 10.21956/wellcomeopenres.20750.r61483

**Published:** 2023-07-18

**Authors:** Gabriela Montejo-Kovacevich

**Affiliations:** 1 University of Cambridge, Cambridge, England, UK

This is a report detailing the sequencing assembly and annotation of *Apamea sordens *or “Rustic Shoulder-Knot” *, *a UK grassland moth. This assembly appears to be of very high quality and the analysis and reporting of the data is well done. It is a very valuable resource for pest research and evolutionary biology.

 The manuscript should be accepted. I only have a couple of small comments:

- It would be helpful if the term ‘moth’ appeared on the title and probably Lepidoptera as well
- Remove colloquial terms that reduce clarity, especially for international readers (e.g. Background “on the wing” to refer to when adults fly)
- The date when the specimen was collected should be specified in the methods, at least the month/year
- I can’t see any files under the genome assembly accession on ENA https://www.ebi.ac.uk/ena/browser/view/PRJEB54051

Are sufficient details of methods and materials provided to allow replication by others?

Yes

Is the rationale for creating the dataset(s) clearly described?

Yes

Are the datasets clearly presented in a useable and accessible format?

Partly

Are the protocols appropriate and is the work technically sound?

Yes

Reviewer Expertise:

Evolutionary biology and genetics

I confirm that I have read this submission and believe that I have an appropriate level of expertise to confirm that it is of an acceptable scientific standard.
